# Supplementary material for: Pericyte heterogeneity identified by 3D ultrastructural analysis of the microvessel wall
Source: Front Physiol. 2022 Dec 16;13:1016382. doi: 10.3389/fphys.2022.1016382 (PMC9800988; doi:10.3389/fphys.2022.1016382)
Supplement: Supplementary file 3 [file DataSheet1.PDF]

SUPPLEMENTAL INFORMATION

Supplemental Movie Legends

Supplemental Movie 1. Animation of a 360° rotation of the 3D rendering for the thin-strand pericyte shown in Figure 1. Blue represents the endothelial cell pseudo-coloring from corresponding SBF-SEM images (reduced opacity), and green represents the pericyte pseudo-coloring.

Supplemental Movie 2. Animation of a 360° rotation of the 3D rendering for the mesh pericyte shown in Figure 1. Blue represents the endothelial cell pseudo-coloring from corresponding SBF-SEM images (reduced opacity), and green represents the pericyte pseudo-coloring.

Supplemental Movie 3. Animation of a 360° rotation of the 3D rendering for the ensheathing pericyte shown in Figure 2. Blue represents the endothelial cell pseudo-coloring from corresponding SBF-SEM images, green represents the pericyte pseudo-coloring, and orange represents the medial cell pseudo-coloring.

Supplemental Movie 4. Animation of a 180° rotation horizontally and vertically of the 3D rendering for the peg-and-socket junction shown in Figure 4. Blue wire-frame represents the endothelial cell pseudo-coloring from corresponding SBF-SEM images, and green represents the pericyte pseudo-coloring.

Supplemental Movie 5. Animation of a 360° rotation of the 3D rendering for the medial cell-endothelial cell junctions (white arrowheads) shown in Figure 5. Blue wire-frame represents the endothelial cell pseudo-coloring from corresponding SBF-SEM images, green represents the pericyte pseudo-coloring, and orange represents the medial cell pseudo-coloring.

Supplemental Movie 6. Animation of a 360° rotation of the 3D rendering for the medial cell-endothelial cell junctions (white arrowheads) shown in Figure 5, and zoomed in from Supplemental Movie 5. Blue wire-frame represents the endothelial cell pseudo-coloring from corresponding SBF-SEM images, green represents the pericyte pseudo-coloring, and orange represents the medial cell pseudo-coloring.
